# Incidence and risk factors of postoperative delirium in the elderly patients with hip fracture

**DOI:** 10.1186/s13018-018-0897-8

**Published:** 2018-07-27

**Authors:** Chen-guang Wang, Ya-fei Qin, Xin Wan, Li-cheng Song, Zhi-jun Li, Hui Li

**Affiliations:** Department of Orthopedics, Tianjin Medical University General Hospital, Tianjin, 300052 People’s Republic of China

**Keywords:** Delirium, Risk factors, Hip facture

## Abstract

**Background:**

To investigate the incidence and related risk factors of delirium in elderly patients with hip fracture.

**Methods:**

This is a retrospective study, performed in a medical center from October 2014 to February 2017, which enrolled all subjects aged over 65 years who were admitted for hip surgeries (hip arthroplasty, proximal femoral nail fixation). Univariate and multivariate logistic analysis was used to determine the incidence and risk factors of delirium. Delirium was assessed according to the Confusion Assessment Method (CAM).

**Results:**

Overall, 19.29% of total 306 patients (mean age 81.9 ± 5.4 years) were identified as delirium. The delirium was significantly associated (*p* < 0.05) with the factors of age, hospitalization, diabetes, preoperative hematocrit (HCT), perioperative protein consumption, transfusion volume, preoperative leukocyte level, albumin level, American Society of Anesthesiologists (NYHA) classification, American Society of Anesthesiologists (ASA) classification, blood loss, coronary heart disease, and cerebral infarction. Multivariate analysis of the variables confirmed that age (> 75 years old), diabetes, and ASA classification (> 2 level) are the independent risk factors of postoperative delirium (POD). In addition, patients in delirium had prolonged hospitalization and high perioperative albumin infusion.

**Conclusion:**

The elderly patients over the age of 75 years with the history of diabetes or ASA classification > 2 level were at higher risk of POD. Delirium is an important postoperative complication, which had prolonged hospitalization and high perioperative albumin infusion.

**Level of evidence: III:**

## Background

Delirium, or acute cognitive function state, is a common postoperative complication that is manifested by a change of mindset and attention deficit over time [1]. Previous literature has described that the incidence of delirium in hospitalized patients vary from 11 to 42% according to population studied [2]. However, the incidence of delirium is 51% following orthopedic surgery for hip fractures. Moreover, in ICU, up to 81% of patients manifest delirium [3]. Postoperative delirium (POD) is associated with extended lengths of stay, higher patient care costs, increased morbidity, and functional and cognitive decline [4]. POD has been reported to be associated with a large number of risk factors: age, dementia, impaired left ventricular function, electrolyte disorder, alcoholism, smoking, high perioperative transfusion requirements, intraoperative pressure fluctuation, and use of benzodiazepine [5–9]. POD occurs mostly in some types of surgery, such as hip surgery, major gastrointestinal surgery, and major cardiac surgery [10–12]. The occurrence and development of delirium following the hip surgery in the elderly is not conducive to the early functional exercise and rehabilitation process [13, 14].The preoperative assessment of the risk factors for delirium is one of the ways to clarify the pathogenesis of POD and propose effective prevention measures. This study focused on elderly patients (aged 65 years or more) admitted to a hospital for conditions (including femoral neck fracture, intertrochanteric fracture) and general surgical procedures (hip arthroplasty, proximal femoral nail fixation). Univariate and multivariate logistic analysis was used to determine the incidence and risk factors of delirium and provide a reliable and accurate theoretical basis for the prevention and treatment of POD.

## Methods

### Patients and design

**Fig. 1:**
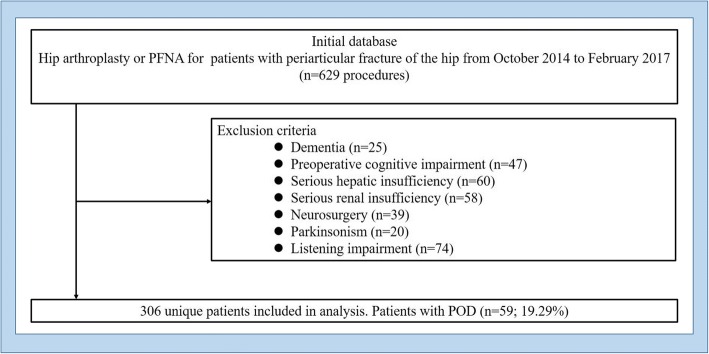
Flow chart of the specific contentᅟ

After obtaining approval from Tianjin Medical University General Hospital Ethics Review Board, we retrospectively reviewed the medical records of all patients aged over 65 years who underwent hip surgery. Thus, patient’s information described in this article was obtained from medical records. Inclusion criteria were as follows: (I) hip fracture (femoral neck fracture, intertrochanteric fracture), (II) the elderly aged over 65 years old, and (III) elective surgery (hip arthroplasty, proximal femoral nail fixation). Exclusion criteria were as follows: (I) preoperative history of schizophrenia, epilepsy, parkinsonism, dementia, delirium, brain injury, or neurosurgery; (II) communication and listening impairment; (III) serious hepatic insufficiency (Child-Pugh class C), serious renal insufficiency (undergoing dialysis before surgery), and hemopathy (leukemia, lymphoma, aplastic anemia); and (IV) preoperative cognitive impairment. The specific content is displayed in the flow chart Fig. 1. Of the patients who participated, the relevant preoperative intraoperative and postoperative demographic and clinicopathologic parameters were recorded. The medical history including cerebral infarction, coronary heart disease, hypertension, diabetes, and the use of benzodiazepines, hypnotics, narcotic drugs, and antiarrhythmic was collected. The suspicious symptoms of POD were evaluated daily after operation by a nerve physician and two trained nurses according to Confusion Assessment Method (CAM). The CAM instrument, which can be completed in less than 5 min, consists of nine operationalized criteria from the Diagnostic and Statistical Manual of Mental Disorders (DSM-HI-R). The diagnosis of delirium by CAM requires the presence of features 1 and 2 and either 3 or 4. The specific content is presented in Table 1.

**Table 1:** Specific content of the Confusion Assessment Method

The Confusion Assessment Method (CAM)	
Feature 1	Acute change in mental status with a fluctuating course
	Is there evidence of an acute change in mental status from the patient’s baseline? Did this behavior fluctuate during the past day, that is, tend to come and go or increase and decrease in severity? This feature is usually obtained from a family member or nurse and is shown by positive responses.
Feature 2	Inattention
	Dose the patients have difficulty focusing attention, for example, being easily distractible, or having difficulty keeping track of what was being said?
Feature 3	Disorganized thinking
	This feature is shown by a positive response to the following question: Was the patient’s thinking disorganized or incoherent, such as rambling or irrelevant conversation, unclear or illogical flow of ideas, or unpredictable switching from subject to subject?
Feature 4	Altered level of consciousness
	Overall, how would you rate this patient’s level of consciousness? (alert [normal], vigilant [hyperalert], lethargic [drowsy, easily aroused], stupor [difficult to arouse], or coma [unarousable])

The diagnosis of delirium by CAM requires the presence of features 1 and 2 and either 3 or 4

### Collection of risk factors

The preoperative risk factors include age, gender, body mass index (BMI), cerebral infarction, coronary heart disease, hypertension, diabetes, American Society of Anesthesiologists (NYHA) classification, Anesthesiologists (ASA) classification, deep venous thrombosis (DVT), use of benzodiazepines, hypnotics, narcotic drugs, antiarrhythmic, hematocrit (HCT), leucocyte count, albumin, total protein (TP) and hemoglobin (Hb), and preoperative hospitalization.

The intraoperative risk factors include duration of operation and anesthesia, type of surgery and anesthesia, blood transfusion volume, and blood loss.

The postoperative risk factors include protein consumption, hematocrit, leucocyte count, hospitalization, hemoglobin, sodium, and potassium.

### Statistical analysis

We use SPSS Statistical software (SPSS, Inc., Chicago, IL) to perform the statistical analysis. Unless otherwise indicated, data are reported as the number of events and their percentage for frequency data and as mean and SD for continuous data. Group comparisons were analyzed by Student’s *t* test for frequency data and the chi-squared test for ordered categorical data. Univariate logistic analysis was used to identify the risk factors associated with POD. The variables identified as xsignificant in univariate analysis were subsequently included in a stepwise multivariate logistic analysis to identify independent predictors of delirium. A *p* value < 0.05 was considered statistically significant.

## Results

### Incidence of postoperative delirium (POD)

Based on the preset inclusion and exclusion criteria, 629 individuals were enrolled from the inception of October 2014 to February 2017 and 323 patients were excluded. The non-delirium group comprised 247 cases, and the delirium group comprised 59 cases. The incidence of POD was 59 (19.29%), including 23 males and 36 females, ranging in age from 70 to 93 years with an average of 81.9 ± 5.4 years old. Two hundred forty-seven cases had no delirium, including 81 males and 166 females, ranging in age from 65 to 96 years, with an average 76.4 ± 8.1 years old.

### Comparison between the two groups

Patients with POD were assigned to the delirium group, and the patients without delirium were assigned to non-delirium group. We compared the various factors between the two groups (Table 2). There were significant differences between the two groups in diabetes, cerebral infarction, coronary heart disease, duration of hospitalization, preoperative hospitalization, preoperative hematocrit, postoperative albumin consumption, blood transfusion, preoperative albumin, preoperative and postoperative leucocyte count, ASA classification, NYHA classification.

**Table 2:** Patient’s clinical characteristics

Variable	Delirium (*n* = 59)	Non-delirium (*n* = 247)		
Age	81.9 ± 5.4	76.4 ± 8.1	*t* = − 0.647	*p* = 0.000
Gender(M/F)	23/36	81/166	*χ*^2^ = 0.813	*p* = 0.367
Hypertension (*n*)	27	147	*χ*^2^ = 3.672	*p* = 0.550
Coronary heart disease (*n*)	35	187	*χ*^2^ = 6.421	*p* = 0.011
Diabetes (*n*)	36	192	*χ*^2^ = 7.006	*p* = 0.008
Cerebral infarction (*n*)	44	214	*χ*^2^ = 5.240	*p* = 0.020
Hospitalization	24.2 ± 15.1	16.0 ± 7.2	*t* = − 4.102	*p* = 0.000
Preoperative hospitalization	5.5 ± 3.4	3.7 ± 1.7	*t* = − 3.677	*p* = 0.000
Albumin infusion	16.7 ± 33.1	1.8 ± 7.1	*t* = − 3.449	*p* = 0.000
Preoperative HCT	33.5 ± 4.9	35.2 ± 5.2	*t* = 2.327	*p* = 0.021
Postoperative HCT	28.2 ± 4.9	29.4 ± 4.8	*t* = 1.641	*p* = 0.102
BMI	22.2 ± 3.2	22.7 ± 3.2	*t* = 1.080	*p* = 0.281
Blood transfusion volume	1.4 + 2.1	0.5 ± 1.0	*t* = − 3.296	*p* = 0.002
Preoperative leucocyte count	9.6 ± 2.6	8 ± 2.1	*t* = − 4.072	*p* = 0.000
Preoperative Hb	11.3 ± 1.6	12.0 ± 5.5	*t* = 0.840	*p* = 0.402
Preoperative albumin	36.5 ± 3.7	37.8 ± 4.0	*t* = 2.132	*p* = 0.034
Preoperative TP	64.6 ± 4.8	64.7 ± 5.8	*t* = 0.046	*p* = 0.963
NYHA	2.2 ± 0.7	1.8 ± 0.6	*t* = − 3.606	*p* = 0.001
ASA	2.9 ± 0.5	2.4 ± 0.5	*t* = − 7.713	*p* = 0.000
Anesthesia	25/34	126/121	*χ*^2^ = 1.422	*p* = 0.233
Surgery	30/29	114/133	*χ*^2^ = 0.421	*p* = 0.516
Operation time	88.5 ± 40.3	79.0 ± 24.3	*t* = − 1.733	*p* = 0.088
The anesthesia time	157.7 ± 50.9	150.3 ± 33.4	*t* = − 1.066	*p* = 0.290
Intraoperative blood loss	127.1 ± 95.2	105.1 ± 33.4	*t* = − 1.666	*p* = 0.046
Postoperative Na+	139.0 ± 4.7	139.6 ± 4.0	*t* = 0.922	*p* = 0.357
Postoperative K+	4.3 ± 0.5	4.3 ± 0.4	*t* = 0.309	*p* = 0.758
Postoperative leucocyte count	9.6 ± 2.6	8.6 ± 2.4	*t* = − 2.717	*p* = 0.007
Postoperative Hb	9.4 ± 1.9	9.7 ± 1.6	*t* = 1.237	*p* = 0.217
DVT (*N*)	15	52	*t* = 0.523	*p* = 0.466

*ASA* American Society of Anesthesiologists, *NYHA* New York Heart Association, *BMI* body mass index, *DVT* deep venous thrombosis, *HCT* hematocrit, *Hb* hemoglobin, *TP* total protein, *F* female, *M* male, *N* numbers

### Logistic regression analysis

Univariate logistic analysis showed that five factors including age (OR 1.090 95% CI 1.021–1.163 *p* = 0.009), diabetes (OR 0.330 95% CI 0.132–0.822 *p* = 0.017), duration of hospitalization (OR 1.106 95% CI 1.053–1.162 *p* = 0.000), ASA classification (OR 4.735 95% CI 1.975–11.355 *p* = 0.000), and albumin infusion (OR 1.051 95% CI 1.008–1.096 *p* = 0.020) are associated with POD (Table 3). The remaining risk factors were not significant in univariate logistic analysis. The result of five factors identified as significant in univariate analysis were included into multivariate analysis. The results indicate that age (OR 1.082 95% CI 1.026–1.140 *p* = 0.003), ASA classification (OR 3.022 95% CI 1.409–6.479 *p* = 0.004), and diabetes (OR 1.041 95% CI 1.009–1.075 *p* = 0.011) were independent risk factors for POD in elderly patients (Table 4). In addition, delirium was associated with prolonged hospitalization and high perioperative protein consumption.

**Table 3:** Univariate analysis of the variables

Variable	*B*	SE	Wals	df	*p* valve	OR	95% CI for exp (B) OR	
							Lower	Upper
Age	0.086	0.033	6.727	1	0.009	1.090	1.021	1.163
Hospitalization	0.101	0.025	15.958	1	0.000	1.106	1.053	1.162
Gender	0.207	0.454	0.207	1	0.649	1.229	0.505	2.992
Hypertension	0.388	0.454	0.730	1	0.393	1.474	0.605	3.590
Diabetes	− 1.110	0.466	5.664	1	0.017	0.330	0.132	0.822
Coronary heart disease	0.408	0.505	0.652	1	0.419	1.504	0.559	4.408
Cerebral infarction	0.448	0.603	0.551	1	0.458	1.564	0.480	5.099
Albumin infusion	0.050	0.021	5.411	1	0.020	1.051	1.008	1.096
Preoperative Hospitalization	0.146	0.093	2.440	1	0.118	1.157	0.964	1.388
Preoperative HCT	− 0.015	0.068	0.047	1	0.828	0.985	0.863	1.125
Postoperative HCT	− 0.022	0.81	0.073	1	0.787	0.978	0.835	1.146
BMI	− 0.085	0.072	1.398	1	0.237	0.919	0.799	1.057
Blood transfusion volume	− 0.172	0.182	0.892	1	0.345	0.842	0.589	1.203
Preoperative leucocyte count	0.163	0.100	2.267	1	0.105	1.177	0.967	1.432
Preoperative albumin	− 0.050	0.079	0.401	1	0.526	0.951	0.815	1.110
Preoperative TP	− 0.016	0.051	0.105	1	0.746	0.984	0.890	1.087
NYHA	0.543	0.323	2.825	1	0.093	1.722	0.914	3.245
ASA	1.555	0.446	12.141	1	0.000	4.735	1.975	11.355
Anesthesia	− 0.107	0.462	0.054	1	0.817	0.899	0.363	2.224
Operation	− 0.111	0.479	0.053	1	0.817	0.895	0.350	2.287
The operation time	0.014	0.013	1.162	1	0.281	1.014	0.989	1.040
The anesthesia time	0.016	0.010	2.511	1	0.113	0.984	0.965	1.004
Intraoperative blood loss	0.006	0.003	3.522	1	0.061	1.006	1.000	1.013
Postoperative Na+	0.063	0.055	1.313	1	0.252	0.939	0.843	1.046
Postoperative K+	0.038	0.449	0.007	1	0.932	0.962	0.399	2.321
Postoperative leucocyte count	0.007	0.099	0.004	1	0.947	1.007	0.829	1.223
Postoperative Hb	0.160	0.224	0.515	1	0.473	1.174	0.758	1.820
DVT	0.338	0.516	0.428	1	0.513	1.402	0.509	3.856

*ASA* American Society of Anesthesiologists, *NYHA* New York Heart Association, *BMI* body mass index, *DVT* deep venous thrombosis, *HCT* hematocrit, *Hb* hemoglobin, *TP* total protein, *CI* confidence interval, *OR* odds ratio, *SE* standard error

**Table 4:** Multivariate analysis of the variables

Variable	*B*	SE	Wals	df	*p* valve	OR	95% CI for exp (B) OR	
							Lower	Upper
Age	0.079	0.027	8.546	8.546	0.003	1.082	1.026	1.140
Hospitalization	0.101	0.022	21.712	21.712	0.000	1.106	1.060	1.154
Diabetes	0.041	0.016	6.409	6.409	0.011	1.041	1.009	1.075
Protein consumption	1.686	0.369	20.823	20.823	0.000	5.396	2.616	11.131
ASA	1.106	0.389	8.077	8.077	0.004	3.022	1.409	6.479
Anesthesia	0.079	0.027	8.546	8.546	0.003	1.082	1.026	1.140

*ASA* American Society of Anesthesiologists, *CI* confidence interval, *OR* odds ratio, *SE* standard error

## Discussion

Delirium is an important postoperative complication which can cause delayed recovery, prolonged hospitalization, and the waste of medical resources [15]. The incidence of POD in our research was 19.29% compared with the incidence rate of 13 to 48% reported by other research [6, 16]. Inouye SK reported that the occurrence of delirium following hip surgery is 12–51% [3]. A variety of diagnostic criteria may be the cause of a significant difference in the incidence of POD. A review of 25 studies showed that 11 instruments have been used to identify the delirium and the CAM was the best choice [17]. Furthermore, small simple size, inclusion criteria, surgery procedures, and anesthesia may lead to variations in incidence rates and risk factors [18, 19]. In our study, delirium was identified by nerve physician according to the evaluation tool of CAM. These measures were designed to ensure the accuracy and integrity of the study.

Although a number of theories have been proposed in an attempt to explain the processes leading to the development of delirium, there is no effective way for the prevention or treatment of POD [20]. The comprehensive assessment of the risk factors for delirium can improves the preventive measures. Many factors were supposed to be associated with POD, such as anesthesia, intraoperative blood loss, blood transfusion, malnutrition, electrolyte disorder high perioperative transfusion requirements, intraoperative pressure fluctuation, and use of benzodiazepine [5, 21, 22]. The results of our research indicate that age (> 75 years old), diabetes, and ASA classification (> 2 level) were strongly independently associated with POD.

Advanced age was consistently considered to be an overlapping risk factor in a review of 80 primary data collection studies by Dyer et al. [23, 24]. Previous studies showed that the occurrence rate of POD increased by 2% when the age of the patient increased by 1 year [4, 25]. Wang et al. [5] believe that the incidence of postoperative delirium in patients aged 70~79 years and over 80 years was higher than that in patients under 70 years old, and the odds ratio (OR) values were 6.33 and 26.37 respectively. Consistent with previous reports, our study demonstrated that the elderly patients over the age of 75 years is the independent risk factors of POD. Older patients are thought to be more susceptible because of the association between aging and the impaired physiologic compensatory capability to adjust to the physical stress of surgery [26]. The changes in the content of central neurotransmitters such as acetylcholine, norepinephrine, epinephrine, and gamma aminobutyric acid are an important cause of delirium as the age increases [27, 28]. In addition, the sensitivity of various mechanisms of blood pressure regulation in the elderly is reduced, so hypotension is easily induced in the induction period. Prolonged hypotension leads to low cerebral perfusion, cerebral ischemia, hypoxia, impairment of brain function, metabolic disorder, disorder of orientation, hallucination, irritability, etc. Edlund et al. [29] found that hypotension within a period of time (systolic pressure of < 100 mmHg) was an independent risk factor for POD and combined epidural anesthesia could lead to a decrease of at least 30% of the blood pressure in the hip joint. The mean arterial pressure fluctuated 30% relative to baseline level when the duration of hypotension lasted for 1 min, which means that the risk of stroke increased by 1.3% [30]. However, Steve [26] believes that age was not an independent risk factor for POD. One reason that age was not significant in multivariate analysis could be that this study dichotomized the age group (i.e., >75 years), while our study use yearly increments in multivariate analysis.

Diabetes is thought to increase the risk of dementia and mild cognitive impairment, as well as an accelerated cognitive decline [31] The cause of susceptibility to delirium in diabetic patients is that there is a general change in the microvascular structure of the brain: the decrease in the number of capillaries, the thickening of the basement membrane, and the increase of the arteriovenous short circuit, which makes the brain tissue more vulnerable to hypoxic damage when the perfusion pressure drops or the blood flow is not smooth [32]. The evaluation of cerebral blood flow and radiation-activity ratio of brain tissue by SPECT indicated that the decrease of senile cerebral blood flow is aggravated by hyperglycemia [33, 34]. A meta-analysis of 14 studies demonstrates that diabetic patients are more prone to cognitive dysfunction [22]. Our research shows that diabetes is associated with an increased incidence of POD, which is consistent with a prospective observational study conducted by Sabol [35], while a single systematic review concluded that diabetes was unrelated to cognitive dysfunction. Reasons for disparity from our findings are that true effect sizes for the association of diabetes with cognitive dysfunction may have been underestimated. Patients with undiagnosed diabetes were included in the respective “no diabetes” groups while the patients with confirmed diabetes were included in our study [36].

The result of our research shows that ASA classification (> 2 level) was strongly independently associated with POD. Several studies have shown that the ASA status is associated with an impaired general physical status and multiple comorbidities [37, 38]. The comorbidities such as diabetes, hypertension, and preoperative cognitive impairment have been previously proved to be the risk factors of delirium in elderly patients [24, 25], and our results corroborated this finding. Multiple comorbidities probably increase baseline vulnerability in older adults, contributing to POD, if combined with other precipitating factors such as major hip surgery. However, Brouquet et al. [39] believe ASA classification (> 3 level) is more likely to lead to POD in elderly patients undergoing major abdominal surgery. The reason may be due to the difference of age criteria for inclusion in the population. All consecutive patients aged 75 years or more were included in Brouquet’s study, while we focused on elderly patients aged 65 years or more.

A practical important finding of our research is that patients in delirium group need more protein consumption during the perioperative period. It accords well with the researches indicating that postoperative delirium is associated with malnutrition and nutritional supplementation could reduce the occurrence of delirium and mortality in the duration of acute trauma and after 4 months [40–42].

To sum up, we recommend that preoperatively comprehensive, accurate management of elderly patients with diabetes and comorbidity could reduce the happening of POD. Furthermore, malnutrition intervention including protein application is beneficial to recovery of patients in delirium group.

This study has some limitations. First, the selected two common types of hip surgery cannot represent the incidence of POD across all kinds of orthopedic surgery. Second, case collection does not follow the principle of randomization. Third, there are misdiagnoses for POD even when medical records were carefully checked.

## Conclusion

The elderly patients over the age of 75 years with the history of diabetes or ASA classification > 2 level were at higher risk of POD. Delirium is an important postoperative complication, which had prolonged hospitalization and high perioperative albumin infusion.

## Abbreviations

ASA: American Society of Anesthesiologists
BMI: Body mass index
DSM-HI-R: Diagnostic and Statistical Manual of Mental Disorders
DVT: Deep venous thrombosis
Hb: Hemoglobin
HCT: Hematocrit
NYHA: New York Heart Association
OR: Odds ratio
POD: Postoperative delirium
SD: Standard derivation
TP: Total protein
